# Characterization of the Meal-Stimulated Incretin Response and Relationship With Structural Brain Outcomes in Aging and Alzheimer’s Disease

**DOI:** 10.3389/fnins.2020.608862

**Published:** 2020-11-30

**Authors:** Jill K. Morris, Casey S. John, Zachary D. Green, Heather M. Wilkins, Xiaowan Wang, Ashwini Kamat, Russell S. Swerdlow, Eric D. Vidoni, Melissa E. Petersen, Sid E. O’Bryant, Robyn A. Honea, Jeffrey M. Burns

**Affiliations:** ^1^Department of Neurology, University of Kansas Medical Center, Kansas City, KS, United States; ^2^University of Kansas Alzheimer’s Disease Center, Kansas City, KS, United States; ^3^Department of Family Medicine, University of North Texas Health Science Center, Fort Worth, TX, United States; ^4^Institute for Translational Research, University of North Texas Health Science Center, Fort Worth, TX, United states; ^5^Department of Pharmacology and Neuroscience, University of North Texas Health Science Center, Fort Worth, TX, United States

**Keywords:** insulin, PYY, Alzheimer’s disease, glucose, insulin resistance, neuroimaging, MRI, voxel based morphometry

## Abstract

**Background:**

Individuals with Alzheimer’s Disease (AD) are often characterized by systemic markers of insulin resistance; however, the broader effects of AD on other relevant metabolic hormones, such as incretins that affect insulin secretion and food intake, remains less clear.

**Methods:**

Here, we leveraged a physiologically relevant meal tolerance test to assess diagnostic differences in these metabolic responses in cognitively healthy older adults (CH; *n* = 32) and AD (*n* = 23) participants. All individuals also underwent a comprehensive clinical examination, cognitive evaluation, and structural magnetic resonance imaging.

**Results:**

The meal-stimulated response of glucose, insulin, and peptide tyrosine tyrosine (PYY) was significantly greater in individuals with AD as compared to CH. Voxel-based morphometry revealed negative relationships between brain volume and the meal-stimulated response of insulin, C-Peptide, and glucose-dependent insulinotropic polypeptide (GIP) in primarily parietal brain regions.

**Conclusion:**

Our findings are consistent with prior work that shows differences in metabolic regulation in AD and relationships with cognition and brain structure.

## Introduction

Insulin resistance and Type 2 Diabetes (T2D) increase with age, and over 60% of older adults ( > 65 years) in the United States exhibit impaired fasting glucose or T2D (Cowie et al., 2006). These conditions are also known risk factors for Alzheimer’s Disease (AD) (Leibson et al., 1997; Ott et al., 1999; Stewart and Liolitsa, 1999; Peila et al., 2002; Arvanitakis et al., 2004; Janson et al., 2004; Yaffe et al., 2004; van der Heide et al., 2006a; Luchsinger et al., 2007; Xu et al., 2009; Profenno et al., 2010; Cheng et al., 2011). However, insulin resistance is also related to dysfunction in a broader, integrated network of metabolic hormones beyond insulin, including peptide tyrosine tyrosine (PYY), glucagon like peptide-1 (GLP-1), and glucose-dependent insulinotropic polypeptide (GIP). These peptides are released by the gastrointestinal tract to stimulate the insulin response and control blood glucose regulation. Their effects are critically important because high glucose levels and impaired glucose regulation are associated with increased AD clinical progression and markers of AD neuropathology (Morris et al., 2014a, b; Macauley et al., 2015).

Although PYY, GLP-1 and GIP are secreted peripherally from the gastrointestinal tract, they cross the blood-brain barrier (Banks and Kastin, 1998; Kastin et al., 2002; Nonaka et al., 2003; Dogrukol-Ak et al., 2004) and have receptors in many brain regions, including those involved in the metabolic response and affected in AD, such as the hypothalamus, temporal and parietal cortex, and hippocampus (Martel et al., 1990a; Usdin et al., 1993; Dumont et al., 1996; Jhamandas et al., 2011). Change in peripheral metabolic hormone secretion thus has the potential to modulate both central nervous system (CNS) and peripheral metabolic function. However, meal-stimulated incretin response has not been compared between cognitively healthy (CH) older adults and those diagnosed with AD.

Older adults with T2D have decreased cross-sectional brain volume (Callisaya et al., 2019), and brain atrophy in T2D individuals may begin as early as midlife (Fang et al., 2018). This suggests that factors related to insulin resistance may be related to brain structure. Given that metabolic hormones are released with each meal and penetrate the brain, it is important to understand these responses in CH older adult and AD populations. The relationship of these responses to brain-relevant outcomes, such as brain structure and cognitive performance, has also never been examined. Thus, the goal of this project was twofold; to characterize the physiological metabolic response to a small mixed meal in cognitively healthy aging and AD, and to determine if these responses track with brain structure and cognition. We also characterized bioenergetic outcomes in platelet mitochondria obtained from these individuals, to further examine differences in energy metabolism. We present here a novel comparison of the incretin response to a mixed meal in CH older adults and individuals with AD, and the relationship of these important metabolic hormones with disease-relevant brain outcomes.

## Materials and Methods

### Participants

All participants in this study provided informed consent according to institutional guidelines and in accordance with the Declaration of Helsinki. Fifty-five participants (*n* = 32 CH, *n* = 23 AD) were recruited by the KU Alzheimer’s Disease Center (KU ADC) recruitment division as previously described (Vidoni et al., 2018). For this study, all enrolled individuals were part of the KU ADC Clinical Cohort and received a comprehensive cognitive and diagnostic evaluation. All participants were evaluated with the Clinical Dementia Rating (CDR) (Hughes et al., 1982; Morris, 1993) and a standard physical and neurological examination using UDS 3.0 Forms and Scales. The UDS 3.0 neuropsychological test battery was then administered.

A weekly diagnostic consensus conference attended by KU ADC clinicians, nurses, neuropsychologists, and psychometricians was held to classify individuals as cognitively healthy (CH; CDR = 0 without clinically significant cognitive impairment evident on testing or evidence of functional decline), Mild Cognitive Impairment (MCI), or AD by standard criteria (Albert et al., 2011; McKhann et al., 2011). Individuals with MCI were further assigned an etiologic diagnosis (i.e., probable or possible AD, etc.). First, CDR impairment and severity staging was reviewed and finalized by consensus review (without reference to cognitive testing). Available cognitive testing results were then reviewed and additional clinical information considered to arrive at consensus on the classification (CH, MCI, AD) and etiologic diagnosis. For this study, all participants met criteria for either etiologic diagnosis of probable AD (any age, CDR 0.5 or 1) or were CH (60 years and older). Exclusion criteria were neurological disease or condition other than AD that may affect cognition (e.g., stroke, major depression, etc.), history of cancer within the last 5 years (except for non-metastatic basal or squamous cell carcinoma), history of drug/alcohol abuse (DSM-IV criteria) within the last 2 years, diagnosed diabetes, and visual or auditory limitations that will interfere with cognitive assessment. Our data flow process has been previously reported (Graves et al., 2015). This study was approved by the University of Kansas Medical Center’s Institutional Review Board (IRB # 03492).

### Neuropsychometric Assessment

All participants received a cognitive examination consisting of the Uniform Data Set (UDS) version 2.0 (Weintraub et al., 2009). Tests were administered by a trained psychometrician in the non-fasting state within 2 months of their metabolic visit date. We used the UDS 3.0 normative calculator (Weintraub et al., 2018) to compute global normative values for each participant. In addition to the UDS, participants were also evaluated using the Mini Mental State Examination (MMSE).

### Anthropometric Measures and Genotyping

Individuals reported for Visit 1 following an overnight fast. Vital signs were measured after a 5 min rest. We measured height to the nearest whole cm and total body mass using a digital scale accurate to 0.1kg (Seca Platform Scale, model 707) and from these values computed body mass index (BMI). Whole blood was collected for Apolipoprotein epsilon 4 (*APOE4)* genotyping. To determine *APOE* genotypes, frozen whole blood was assessed using a Taqman single nucleotide polymorphism (SNP) allelic discrimination assay (Thermo Fisher Scientific). *APOE ε2*, *ε3*, and *ε4* alleles were distinguished using Taqman probes to the two *APOE*-defining SNPs, rs429358 (C_3084793_20) and rs7412 (C_904973_10). The term “Carrier” is used to describe the presence of 1 or 2 APOE ε4 alleles.

### Meal Tolerance Testing

Following an overnight fast, subjects consumed 1 bottle of Ensure (220 calories, 33g carbohydrates) within 5 min. Blood was collected at 0, 15, 30, 45, 60, 90, and 120 min post-meal into tubes containing EDTA (for glucose, insulin, and C-peptide analyses) or DPP-IV inhibitors (p800 tubes, BD Biosciences) for incretins (GIP, GLP-1, PYY). Plasma glucose was measured using a glucose analyzer (YSI 2300, Yellow Springs Instruments). Plasma insulin and C-Peptide (ALPCO) as well as GIP (IBL) were measured using ELISA. Both GLP-1 and PYY were analyzed using a multi-plex electrochemiluminescent (ECL) assay per previously established methods with commercially available kits (Meso Scale Discovery, MSD) (O’Bryant et al., 2014, 2016). ECL utilizes a label that emits light when electronically stimulated, thus improving sensitivity of detection even at low concentrations. The coefficient of variation (CV) and lowest level of detection (LLOD) are reported for the following MSD assays: GLP-1 (CV = 3.75; LLOD = 0.06 pg/mL) and PPY (CV = 3.39; LLOD = 5.79 pg/mL).

### Platelet Mitochondrial Enzyme Activity Measures

For a subset of subjects [*n* = 40 (*n* = 20 HC and *n* = 20 AD)], platelet mitochondria were isolated from fasted fresh whole blood into acid citrate dextrose tubes at fasting and re-suspended into MSHE buffer. Cytochrome oxidase (COX) and citrate synthase (CS) Vmax activities were assessed spectrophotometrically. For the COX Vmax, we followed the conversion of reduced cytochrome C to oxidized cytochrome c and calculated the pseudo-first order rate constant (ms^–1^). For the CS Vmax, we followed the formation of 5-thio-2-nitrobenzoate (nmol/min). Both rates were normalized to mg total protein (BCA assay).

### Neuroimaging Measures

T1-weighted MPRAGE anatomic images (TR/TE = 2,000/3.06 ms, flip angle = 8°, FOV = 192 × 100 mm, matrix = 192 x 192) were collected on 55 subjects using a 3T Skyra Siemens scanner. Every scan was checked for image and motion artifacts and gross anatomical abnormalities, resulting in the removal of 1 subject, as well as 1 subject who did not have PYY data for analysis, leaving a VBM sample of 53 subjects (31 CH subjects and 22 AD subjects).

For voxel-based morphometry (VBM) analyses and pre-processing of T1-weighted images we used the Computational Anatomical Toolbox 12 (CAT12 Version 12.6, C. Gaser, Structural Brain Mapping Group, Jena University Hospital, Jena, Germany)^1^ through Statistical Parametric Mapping version 12 (SPM12; Wellcome Trust Centre for Neuroimaging, London, United Kingdom)^2^ that operate under Matlab (R2019b) (the Mathworks, Natick, MA) on Mac. T1 images were corrected for bias-field inhomogeneities, registered using linear (12-parameter affine) and non-linear transformations, spatially normalized using the high-dimensional DARTEL algorithm into MNI space (O’Bryant et al., 2016), and segmented into gray matter (GM), white matter (WM), cerebrospinal fluid (CSF) and white matter hyperintensity (WMH). We calculated total intracranial volume (TICV) using total gray, white, and CSF volumes. The amount of volume changes were scaled, in order to retain the original local volumes (modulating the segmentations) (O’Bryant et al., 2016). The modulated gray matter segmentations were smoothed using a 10 × 10 × 10 mm full-width at half-maximum Gaussian kernel prior to group level analysis uses and images from the internet).

### Statistical Analyses

#### Metabolic, Anthropometric, and Cognitive Outcomes

Diagnostic differences were assessed using ANOVA (for continuous variables) or chi square analyses (for categorical variables). Given prior work showing relationships to both metabolic function and AD, all measures were controlled for age, sex, body mass index (BMI), and APOE4 (carrier status). Results were considered significant at *p* < 0.05.

#### Neuroimaging

We used a General Linear Model full factorial analysis with *post-hoc t*-tests to assess the main effect of diagnosis on smoothed normalized gray matter images, including the same covariates as metabolic outcomes, in addition to TICV. In each separate analysis we included the metabolic biomarker response to a mixed-meal measured as area under the curve (AUC) [glucose, insulin, PYY, GIP, GLP1, C-Peptide, and platelet mitochondrial enzyme function (Citrate synthase Vmax)] as a covariate for interaction with diagnosis (see Table 2 for variable details). *Post-hoc* tests also included a combined-group (ND and AD combined) regression of the metabolic biomarkers across gray matter volume with the same covariates as the initial analysis.

For all analyses, voxels are reported with reference to the MNI standard space within SPM12. To avoid possible edge effects at the border between GM and WM and to include only relatively homogeneous voxels, we used an absolute threshold masking of 0.10 for each analysis. Results for *f*-tests and *t*-tests were considered significant at *p* < 0.05 after correction for multiple comparisons (family-wise error, FWE), and results at *p* < .001 for *t*-tests are shown in Supplementary Table 1, with a minimum cluster size of 100 voxels (*k* > 100) for all analyses. After whole brain unmasked analysis, we used a small volume correction (SVC) to test for associations between the overall meal response (AUC) variables and two anatomical mask regions, one encompassing regions involved in eating and reward behavior (Batterham et al., 2007; Neary and Batterham, 2010; Weise et al., 2012), and a second combining regions involved in the default-mode network (DMN), where we have previously observed relationships between glucose metabolism and amyloid neuropathology (Morris et al., 2016b; Taylor et al., 2017). The mask for food regions included the caudate nucleus, globus pallidus, thalamus, prefrontal regions, anterior cingulate gyrus and the cerebellum and was created by combining these regions into a single anatomical masks using the WFU pickatlas tool (Maldjian et al., 2003) and the integrated automatic anatomic labeling (AAL) tool (Tzourio-Mazoyer et al., 2002). The mask for the DMN network included the hippocampus, parahippocampal gyrus, amygdala, anterior cingulate gyrus, superior medial frontal cortex, precuneus, inferior parietal lobe, superior parietal lobe, and the posterior cingulate (Buckner et al., 2008).

## Results

### Subject Characteristics

CH and AD diagnosis groups did not differ by age, sex, BMI, body weight, APOE4 carrier status, education or blood pressure (Table 1). As expected, individuals with AD were characterized by lower MMSE scores and higher CDR-Sum of Boxes compared to CH older adults.

**TABLE 1:** Subject characteristics.

Measure	CH (*n* = 32)	AD (*n* = 23)	*p*-value
Age (year)	74.0 (5.4)	76.3 (6.3)	0.161
Sex (#, % male)	14 (42.4)	12 (52.2)	0.470
APOE4 (#, % carrier)	12 (36.4)	12 (54.5)	0.180
BMI	28.1 (4.3)	27.7 (5.4)	0.746
Education (y)	15.9 (2.8)	15.6 (3.0)	0.719
Systolic BP (mm/Hg)	135.7 (16.3)	130.6 (12.2)	0.173
Diastolic BP (mm/Hg)	76.2 (8.3)	73.6 (6.3)	0.136
Global cognition (z-score)	0.186 (0.55)	−1.57 (1.1)	<0.01*
Weight (kg)	76.8 (14.1)	77.8 (21.3)	0.740
CDR-SB	0 (0)	4.26 (2.4)	<0.01*
MMSE	29.1 (0.87)	22.7 (5.1)	<0.01*

Groups were well matched based on participant characteristics. As expected, AD subjects had lower cognitive scores. CH, Cognitively Healthy; AD, Alzheimer’s Disease; APOE4, Apolipoprotein epsilon 4; BMI, body mass index; BP, blood pressure; CDR-SB, Clinical Dementia Rating Sum of Boxes; MMSE, mini mental state examination. *p < 0.05. Bold values indicate significant differences.

### Meal Stimulated Response

#### Area-Under-the-Curve (AUC)

**FIGURE 1:**
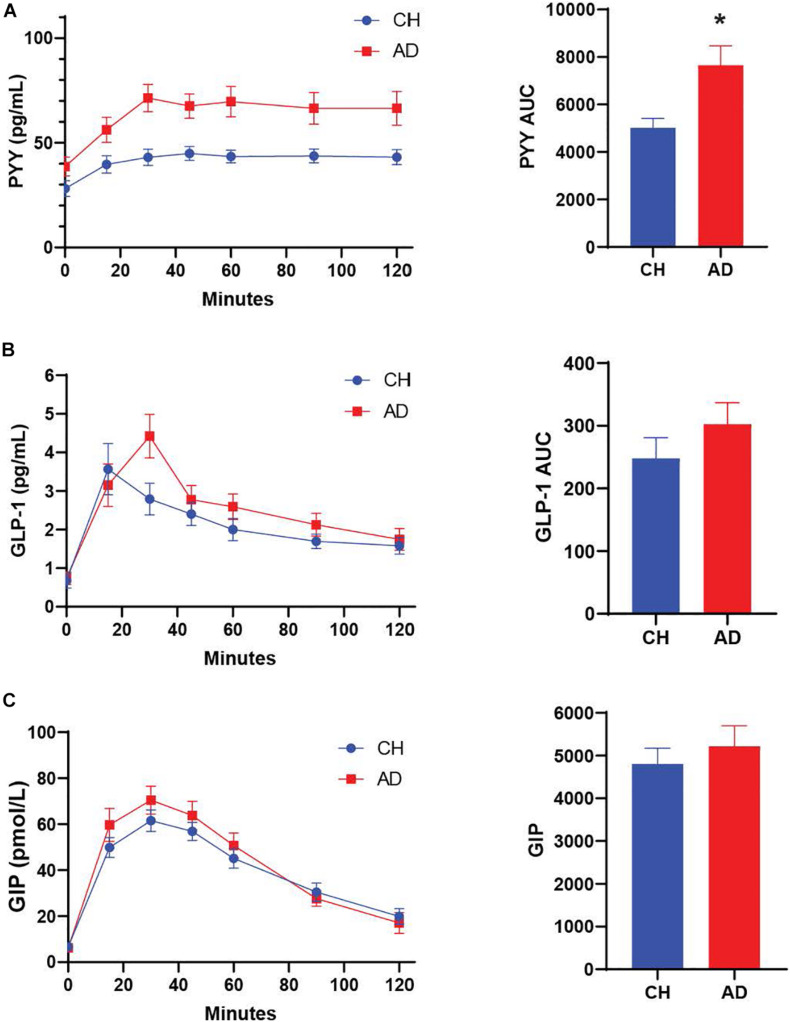
**(A)** PYY response to a mixed meal (AUC 0-120) is significantly higher in AD subjects compared to CH older adults. No significant differences are observed for GLP-1 and GIP **(B,C)** *p < 0.05.

**FIGURE 2:**
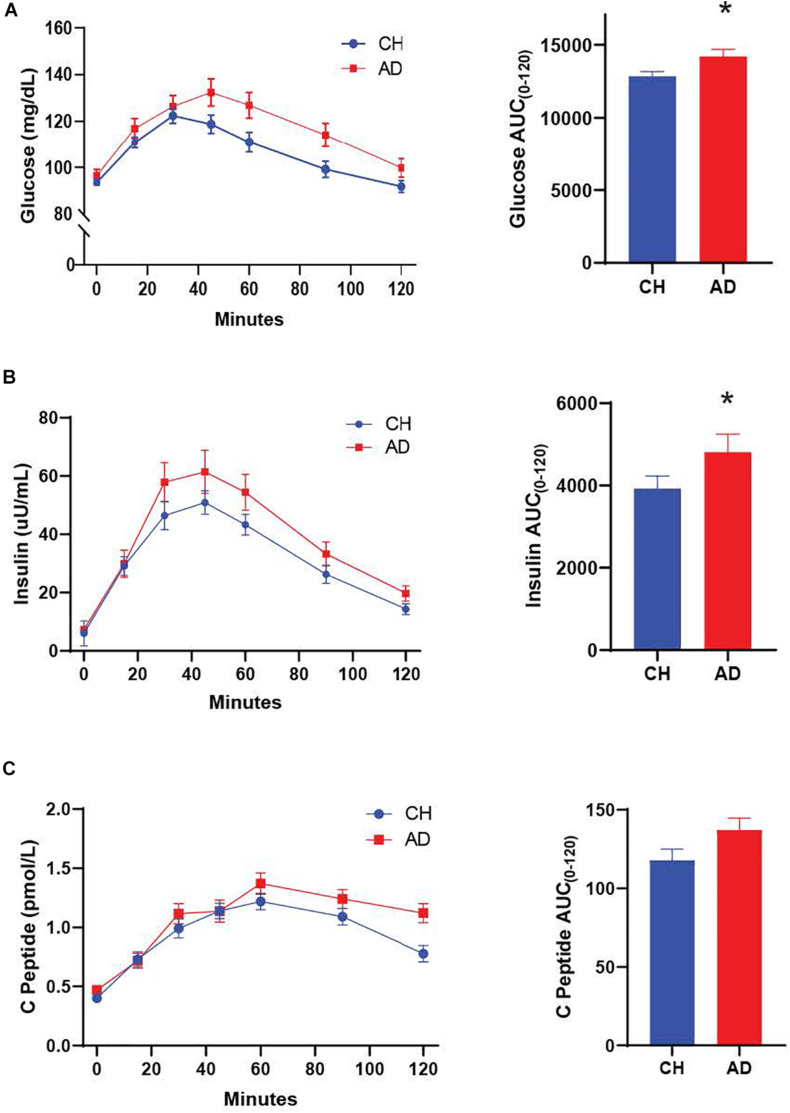
Both glucose **(A)** and insulin **(B)** responses to a mixed meal were elevated in AD subjects. **(C)** The C-Peptide response did not differ between diagnosis groups. *p < 0.05.

We characterized diagnostic differences in the mixed-meal stimulated responses by calculating the AUC for key metabolic hormones. AD subjects exhibited a higher AUC compared to CH individuals in response to a mixed meal for PYY (*p* = 0.001; Figure 1, insulin (*p* = 0.036; Figure 2), and glucose (*p* = 0.035; Figure 2), with no diagnostic differences observed in the response of GLP-1, GIP, or C-Peptide.

#### Early Meal Response

The difference between the fasting and 30 min timepoint of glucose tolerance tests has been used to determine the early meal response (Cozma et al., 2005). This early meal response tracks well with first phase insulin secretion, which is especially important for control of glucose production by the liver (Luzi and DeFronzo, 1989). We calculated the early meal response (Δ0–30) values for these same metabolic biomarkers and identified diagnostic differences for PYY (*p* < 0.001) and GLP-1 (*p* = 0.026), with higher responses in AD individuals (Table 2).

**TABLE 2:** Early response to a mixed meal.

Measure	Cognitively healthy (*n* = 32)	AD (*n* = 23)	*p*-value
Glucose (mg/dL)	29.1 (16.0)	35.7 (14.1)	0.250
Insulin (μU/mL)	40.4 (25.3)	50.6 (29.6)	0.147
C-Peptide (ng/mL)	0.59 (0.33)	0.686 (0.33)	0.347
GIP (pmol/L)	54.9 (26.4)	64.3 (27.9)	0.220
Peptide YY	14.9 (15.2)	32.8 (19.3)	<0.001*
GLP-1	2.11 (1.9)	3.68 (2.7)	0.026*

Early response (Δ0–30) values for bioenergetic outcomes following a mixed meal. GIP, Gastric inhibitory polypeptide; Peptide YY, Peptide Tyrosine Tyrosine; GLP-1, GLP-1, Glucagon-like peptide 1. *p < 0.05. Bold values indicate significant differences.

#### Neuroimaging

**FIGURE 3:**
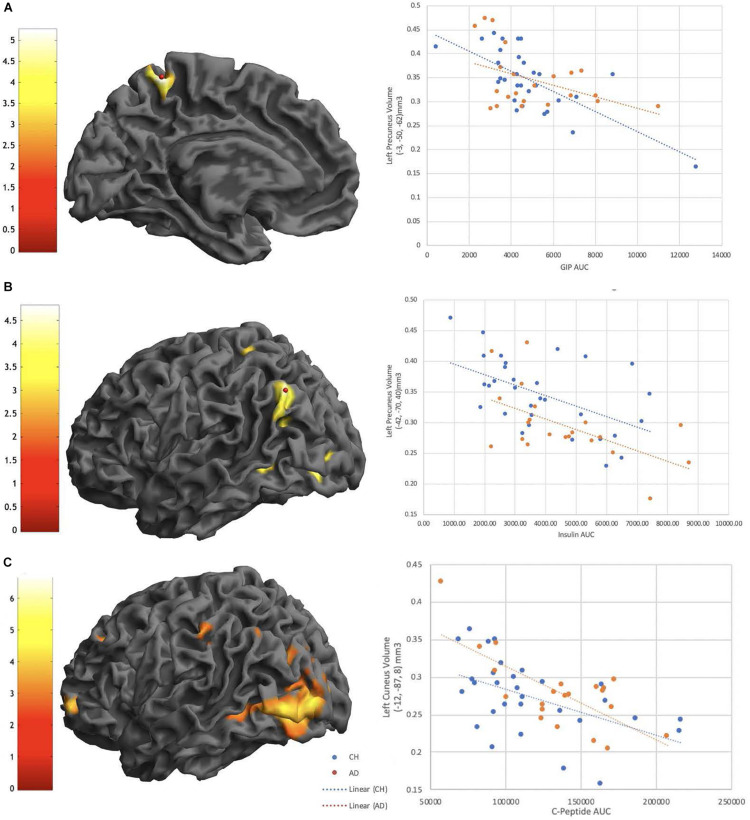
GIP AUC **(A)**, Insulin AUC **(B)**, and C-Peptide AUC **(C)** are significantly negatively correlated with interior left precuneus **(A)**, Left precuneus **(B)**, and Left Cuneus volume **(C)** all in the parietal cortex. Extracted volumes plotted *post-hoc* against hormone AUC in plots on the right.

In the overall cohort there was a significant difference in gray matter volume between diagnosis groups, the AD group had decreased gray matter volume compared to the ND group in a large cluster encompassing the left middle temporal gyrus and right post-central gyrus (Table 3). We identified that Insulin AUC, GIP AUC and C-Peptide AUC all had significant negative relationships with gray matter volume in CH and AD subjects, primarily in the parietal cortices (Figure 3). At a whole brain level, C-Peptide AUC negatively correlated with the left cuneus (*p* < 0.001, *Z* = 5.51, −12, −87, 8) and another cluster in the left parietal lobe seen using the DMN network SVC (*p* < 0.05, *Z* = 4.17, −58, −46, 46) (Table 3). Also at a whole brain level, GIP AUC negatively correlated with the left precuneus (*p* < 0.05, *Z* = 4.57, −3, −50, 62), which also presented significant in the DMN network SVC. In the DMN network SVC Insulin AUC was negatively correlated with the Left Inferior Parietal Lobe (*p* < 0.05, *Z* = 4.27, −42, −72, 40). There were no significant positive relationships with gray matter volume with the metabolic hormones.

**TABLE 3:** Regional imaging relationships.

*F*-test main effect of diagnosis		Peak F	*Z*	Cluster (k)	Peak *p*(FWE-corr)	Peak p(unc)	x,y,z (mm)	Regions
	Whole brain	**47.27**	**5.52**	**2052**	**0.001**	**0.000**	**−63, −46, -9**	**Left middle temporal gyrus**
		**33.82**	**4.85**	**101**	**0.012**	**0.000**	**62, −22. −32**	**Right post-central gyrus**
*T*-test direction specific effects		Peak T	Z	cluster (k)	Peak p (FWE-corr)	Peak p(unc)	x,y,z (mm)	Regions
**Negative regression of insulin AUC and GMV (ND and AD together)**								
	DMN network SVC	**4.80**	**4.27**	**326**	**0.026**	**0.000**	**−42, −72, 40**	**Left inferior parietal lobe**
		3.97	3.65	286	0.207	0.000	45, **−**74, 42	Right angular gyrus
		3.75	3.46	276	0.336	0.000	12, **−**51, 30	c
**Negative regression of GIP AUC and GMV (ND and AD together)**								
	Whole brain	**5.23**	**4.57**	**663**	**0.035**	**0.000**	**−3, −50, 62**	**Left precuneus**
		4.99	4.41	385	0.066	0.000	**−**52, **−**80, 10	Left middle temporal gyrus
		4.08	3.73	229	0.512	0.000	**−**54, **−**40, 51	Left Inferior Parietal Lobe
	DMN network SVC	**5.21**	**4.56**	**250**	**0.008**	**0.000**	**−3, −51, 62**	**Left precuneus**
		4.08	3.73	136	0.158	0.000	**−**54, **−**40, 51	Left Inferior Parietal Lobe
**Negative regression of C-peptide AUC and GMV (ND and AD together)**								
	Whole brain	**6.73**	**5.51**	**6581**	**0.001**	**0.000**	**−12, −87, 8**	**Left cuneus**
						**0.000**	**−22, −96, 0**	**Right cuneus (calcarine)**
						**0.000**	**−38, −84, 0**	**Left middle occipital gyrus**
		4.68	4.17	259	0.155	0.000	**−**58, **−**46, 46	Left Inferior Parietal Lobe
		4.52	4.06	642	0.224	0.000	**−**39, 46, **−**2	Left middle frontal gyrus
		4.50	4.04	1604	0.236	0.000	26, **−**76, 26	Right occipital gyrus
		4.37	3.95	172	0.307	0.000	40, 20, 15	Right inferior frontal gyrus
		4.22	3.83	487	0.416	0.000	64, **−**33, 3	Right superior temporal gyrus
		4.07	3.71	152	0.545	0.000	40, **−**42, **−**14	Right fusiform gyrus
		3.94	3.62	130	0.65	0.000	**−**4, **−**4, 52	Left superior motor area
		3.89	3.57	187	0.698	0.000	**−**15, 36, 34	Left superior motor area
		3.79	3.49	102	0.778	0.000	0, **−**14, 75	Medial frontal gyrus
		3.76	3.47	116	0.802	0.000	40, **−**63, **−**4	Right middle temporal gyrus
	DMN network SVC	**4.68**	**4.17**	**168**	**0.037**	**0.000**	**−58, −46, 46**	**Left parietal lobe (BA 40)**

*Bold values indicate significant differences.*

**FIGURE 4:**
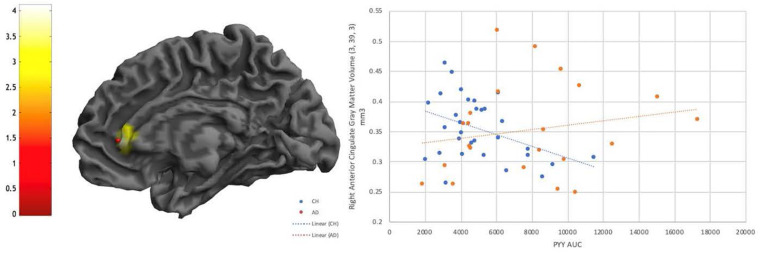
Diagnostic interaction effect is evident for PYY in the Anterior Cingulate, with a negative relationship visible in CH individuals that is not evident in AD. *P* < 0.001 uncorrected.

There were no significant interactions of diagnosis with any of the metabolic hormones at a whole brain level or in the food region or DMN SVC, however, there were several interactive effects that reached a trend level of significance (*p* < 0.001 uncorrected, *k* > 100) (Supplementary Table 1). There was an interactive relationship between PYY and diagnosis such that CH individuals had a more negative relationship between PYY and right anterior cingulate and inferior frontal gyrus than individuals with AD (Figure 4). There were also interactive relationships between Insulin AUC and diagnosis, and similarly in GLP1 and diagnosis, in superior temporal gyrus as well as frontal regions (Supplementary Table 1), as well as a negative relationship between GLP1 AUC and gray matter volume in the right superior temporal gyrus in both diagnosis groups. Finally, there was a positive interactive relationship (greater gray matter volume alongside larger hormone AUC in the AD group compared to CH) between C-Peptide AUC and diagnosis in the right angular gyrus and right inferior parietal lobe.

### Cognitive Performance

All individuals in this study were administered a cognitive battery consisting of the Uniform Data Set Version 2.0 (Weintraub et al., 2009). We computed normative z-scores for each UDS cognitive test as previously described (Weintraub et al., 2018) and well as a z-score for global cognition, which is a mean of all z-scores in the battery (Table 1). In the overall cohort, we observed a significant negative linear relationship between gAUC and the global cognition z-score (β = −0.391, *p* = 0.008). Global cognition did not track with other metabolic biomarkers.

### Platelet Mitochondrial Function

Generation of ATP in mitochondria is coupled to insulin exocytosis, (Maechler and Wollheim, 2001) and insulin secretory granules are in close proximity to mitochondria to promote coupling of metabolism and insulin secretion (Wollheim, 2000). Given the potential impact of mitochondrial dysfunction on insulin dysregulation, we performed functional assessments to characterize the activity of key mitochondrial enzymes in blood platelets to evaluate potential diagnostic differences (Table 1). Cytochrome oxidase activity (maximal velocity; Vmax) was measured and did not differ between diagnosis groups (*p* = 0.583). Citrate synthase (CS) Vmax was also characterized and did not differ between groups in this study (*p* = 0.277). Because the relationship of platelet mitochondrial function and brain structure has never been examined, we also characterized the relationship between these measures and brain structure using VBM. Across diagnosis groups, we observed a positive relationship between CS Vmax and brain volume in the left and right frontal gyrus, as well as the left precuneus (Supplementary Table 1). However, these findings did not hold up to multiple comparisons corrections.

## Discussion

**FIGURE 5:**
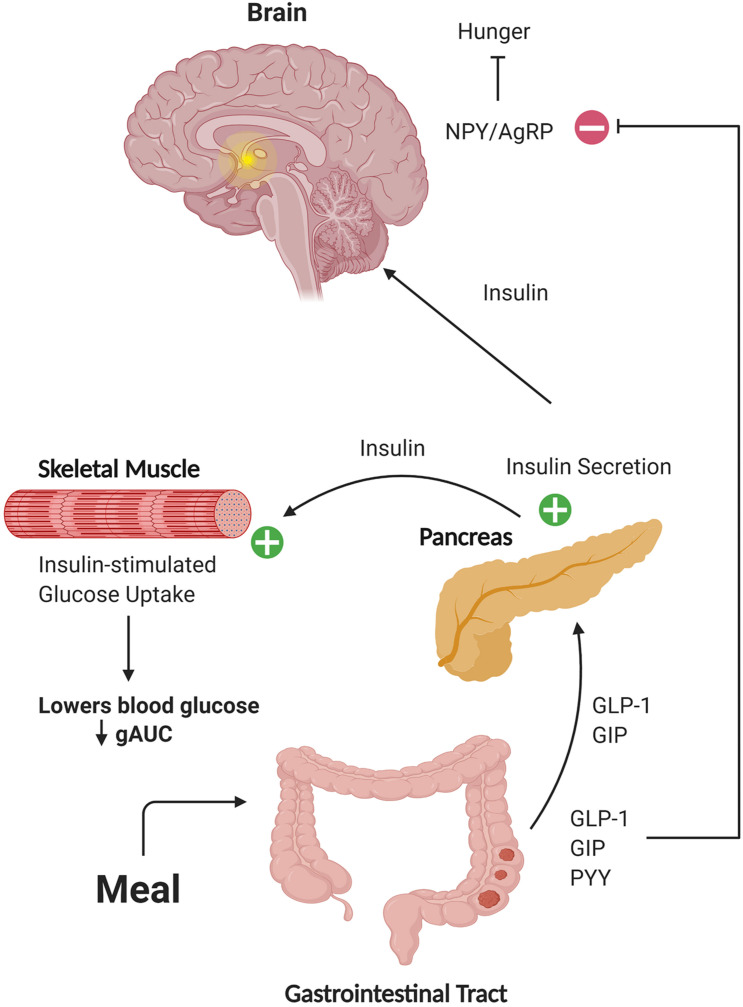
Schematic representation of hormone action on target tissues. Gut-secreted hormones can travel through the circulation to the pancreas and brain. In these tissues, these hormones can potentiate secretion of insulin and affect regulation of blood glucose levels, as well as activate neuronal populations involved in responses such as satiety. Figure created with BioRender.

The hormone insulin has been implicated in neurotransmission and cell survival (Wan et al., 1997; Skeberdis et al., 2001; Uemura and Greenlee, 2006; van der Heide et al., 2006b; Jin et al., 2011), associated with better cognition and less brain atrophy in AD (Burns et al., 2007), and been shown to improve memory in AD when administered intranasally (Reger et al., 2008a, b; Craft et al., 2012). However, insulin-sensitizing agents have not shown cognitive benefit or improved brain glucose metabolism (Gold et al., 2010; Tzimopoulou et al., 2010; Harrington et al., 2011). This may be due in part to inefficient transport of these compounds across the blood-brain barrier, but also suggests that additional mechanisms associated with production of insulin rather than just sensitization may be important. Here, we characterized diagnostic differences in metabolic biomarkers following a mixed meal and identified relationships of these biomarkers with brain structure. We focused on metabolites directly involved in or affected by insulin secretion, including insulin, GIP, GLP-1, PYY, C-peptide, and glucose. This is the first study to examine the response of Peptide YY and revealed striking elevations in AD participants compared to CH subjects. Elevations in the glucose and insulin AUC, which have been previously described as related to structural brain outcomes in other studies of AD subjects (Burns et al., 2007; Burns et al., 2012), were also observed. Although no group differences in the meal-stimulated AUC response were observed for either GIP or C-peptide, significant negative relationships between brain volume and AUC for these hormones, as well as insulin, were observed in highly metabolic brain regions such as the precuneus and parietal lobe across groups. Taken together, our data shows that a variety of diagnostic differences and relationships with structural outcomes are evident with the metabolic hormone response in response to a small (220 calorie) mixed meal. The effects of these peripheral hormone response differences reach far beyond the tissues of origin (Figure 5).

The observed increase in the early meal response of Peptide YY and GLP-1 suggests that in AD subjects, compensatory responses exist to increase insulin levels and maintain normoglycemia. Although both PYY and GLP-1 are stored within enteroendocrine cells, they exist within discrete compartments that allow for differential release (Cho et al., 2014). This may explain the sustained elevation of PYY beyond that of GLP-1. PYY is also produced in pancreatic islet cells, where the full-length peptide can locally inhibit insulin secretion through effects on Y1 receptors, but cleaved PYY activates Y2 receptors and is linked to increased circulating insulin levels, potentially due to simultaneous release of GLP-1 (Persaud and Bewick, 2014). This is worth noting as we did observe early increases in GLP-1 release in AD subjects, although again, this was not sustained throughout the MTT. It is known that plasma PYY levels rise in response to a meal (Adrian et al., 1985), and PYY can freely cross the blood-brain barrier (Batterham and Bloom, 2003). PYY inhibits food intake through actions in the arcuate nucleus of the hypothalamus, but also acts on receptors present in the temporal cortex and hippocampus (Martel et al., 1990b; Roder et al., 1996). While PYY receptor affinity is not altered between AD and CH individuals, PYY receptor density is lower in the hippocampus in AD (Martel et al., 1990a). Thus, it is possible that the increase in PYY release we observed in response to a mixed meal could be a compensatory response due to decreased receptor density in key brain regions. We also observed diagnostic elevations in the glucose and insulin meal responses. Our observation of an elevated glucose response in response to increased insulin and PYY compensation is insufficient to normalize glucose tolerance in these individuals.

We then investigated whether the meal response of these metabolically active peptides (AUC value) tracked with brain structure using VBM. The strongest effects were consistently in the negative direction across both groups, occurred in the parietal cortex (Figure 3), and withstood multiple comparisons correction. Specifically, both insulin and C-Peptide AUC tracked negatively with brain volume in the left inferior parietal cortex (Table 3), while GIP and C-peptide AUC tracked negatively with brain volume in the precuneus and cuneus, respectively (Table 3). Given the role of GIP as an incretin hormone and C-peptide as an insulin cleavage product, these relationships in known highly metabolic brain regions underscore important relationships between insulin and brain structure. Insulin resistance is linked to increased atrophy (Benedict et al., 2012; Willette et al., 2013), and we have shown that prediabetes is related to increased rate of overall brain atrophy over two years in AD (Morris et al., 2016b). We have also shown a relationship between insulin resistance and decreased medial temporal, frontal and occipital cortical volumes (Morris et al., 2014b). These are consistent with our finding that increases in GIP, insulin and C-Peptide were related to decreased volume in regions associated with AD disease pathology, and a growing amount of data are linking insulin and insulin-like growth factor, type 1 (IGF-1) deficiencies to the pathogenesis of AD (de la Monte et al., 2018). It is worth noting that consistent negative relationships with brain volume were also observed with the AUC response of glucose, insulin, GIP, and C-peptide in temporal regions, and although these did not withstand FWE correction (Supplementary Table 1), they were in metabolically sensitive regions consistent with our previous work and will need to be investigated with a larger sample or a larger meal stimulus.

PYY was the only hormone that showed an interaction effect between diagnosis groups (Figure 4), which occurred in the right anterior cingulate and inferior frontal gyrus. In CH older adults, increased PYY was associated with decreased brain volume, but this was not observed in AD subjects. Our findings in CH individuals correspond with results from a previous study on PYY concentrations and brain volume in non-diabetic young adults, where the authors found a relationship with PYY response and anterior cingulate volumes among others (Weise et al., 2012). The anterior cingulate bridges brain regions involved in autonomic function, cognition, and reward processing (Stevens et al., 2011), and many studies have shown decreased functional connectivity with this region in AD compared to healthy controls. This suggests that cognitively healthy individuals who have a compensatory increase in PYY (potentially due to very early stage IR) have lower anterior cingulate brain volume. It is possible that the lack of relationship in AD individuals is due to more heterogeneity in brain volume in this group, which prior work has shown to vary based upon the presence of neuropsychiatric symptoms in AD subjects (Tascone et al., 2017).

Despite the lack of neuroimaging relationships that withstood multiple comparisons corrections compared to the other biomarkers examined, gAUC was the only biomarker that tracked significantly with cognitive performance (global cognition). This suggests that the effect of insulin and related hormones and brain volume was stronger, or that the effect of glucose may be more readily detected using a larger meal stimulus. This finding also underscores the important relationship between glucose regulation and cognitive function previously demonstrated in larger epidemiological studies (Altschul et al., 2018; Zheng et al., 2018), an effect may occur independently of large changes in brain structure.

Our findings build upon prior work from our group and others that suggest insulin dysregulation exists peripherally and centrally in AD (Burns et al., 2007; Craft et al., 2012; Talbot et al., 2012; Morris et al., 2016a), and extends these findings to include additional metabolic-related hormones. Strengths of this study include the robust clinical characterization of our diagnosis groups, which were also well-matched in terms of age, sex, and BMI. An important additional strength is the careful pre-processing of plasma samples for incretin analysis, which were collected in tubes containing dipeptidyl peptidase 4 (DPP-4) inhibitor prior to processing and storage. This is critical for accurate incretin measurement. A limitation of the study is the sample size, which may have limited our ability to detect smaller diagnostic differences in responses, as well as the cross-sectional nature of the study. Additional considerations should include the fasting time, caloric content, and type the meal stimulus, which likely affect the hormone response, as well as potential effects of APOE4 genotype and sex, which should be explored in future studies. It is also important to note that it is unclear how closely our measures of the peripheral hormone response reflect hormone levels in brain. Nonetheless, we provide evidence for diagnostic differences in the response of emerging gut hormones to a small mixed meal. Given that multiple meals and snacks are consumed throughout the day, these differences may have important physiological consequences that become evident over time. Future studies should examine the longitudinal effects of these changes on brain outcomes.

## Data Availability Statement

The raw data supporting the conclusions of this article will be made available by the authors, without undue reservation, to any qualified researcher.

## Ethics Statement

The studies involving human participants were reviewed and approved by the University of Kansas Medical Center Institutional Review Board. The patients/participants provided their written informed consent to participate in this study.

## Author Contributions

JM and JB: study design. JM, CJ, HW, and XW: conducting experiments. JM, CJ, HW, XW, and SO’B: acquiring data. JM, ZG, AK, EV, and RH: analyzing data. JM, CJ, ZG, HW, EV, RS, MP, RS, and JB: manuscript drafting.

## Conflict of Interest

The authors declare that the research was conducted in the absence of any commercial or financial relationships that could be construed as a potential conflict of interest.

## Footnotes

1 http://dbm.neuro.uni-jena.de/cat/

2 http://www.fil.ion.ucl.ac.uk/spm/software/spm12/
