# Supplementary material for: Characterization of the Meal-Stimulated Incretin Response and Relationship With Structural Brain Outcomes in Aging and Alzheimer’s Disease
Source: Front Neurosci. 2020 Nov 30;14:608862. doi: 10.3389/fnins.2020.608862 (PMC7734152; doi:10.3389/fnins.2020.608862)
Supplement: Supplementary file 1 [file Table_1.DOCX]

Supplemental Table 1

| **T-Test** Direction Specific Effects | | Peak T | Z | cluster (k) | Peak p (FWE-corr) | Peak p(unc) | x,y,z (mm) | Regions |
| --- | --- | --- | --- | --- | --- | --- | --- | --- |
| Negative Interaction between PYY AUC and Diagnosis | |  |  |  |  |  |  |  |
|  |  | 4.09 | 3.74 | 407 | 0.495 | 0.000 | 3, 39, 3 | Right Anterior Cingulate (BA 32) |
|  |  | 3.74 | 3.46 | 75 | 0.795 | 0.000 | 50, 30, -15 | Right Inferior Frontal Gyrus |
| Negative Interaction between Insulin AUC and Diagnosis | | |  |  |  |  |  |  |
|  |  | 4.42 | 3.99 | 225 | 0.274 | 0.000 | -44, 6, -26 | Left Superior Temporal Gyrus |
|  |  | 4.08 | 3.73 | 375 | 0.523 | 0.000 | 56, 16, 4 | Right Inferior Frontal Gyrus |
|  |  | 4.05 | 3.71 | 186 | 0.550 | 0.000 | 12, -93, 34 | Right Cuneus (BA 19) |
| Negative Interaction between GLP1 AUC and Diagnosis | | |  |  |  |  |  |  |
|  |  | 4.35 | 3.94 | 135 | 0.305 | 0.000 | -42, -36, -14 | Left Inferior Temporal Gyrus |
|  |  | 4.1 | 3.74 | 165 | 0.497 | 0.000 | -52, -10, 8 | Left Superior Temporal Gyrus |
|  |  | 3.84 | 3.54 | 129 | 0.740 | 0.000 | 3, -8, 56 | Right Medial Frontal Gyrus |
| Negative Interaction between CS Protein and Diagnosis | | |  |  |  |  |  |  |
|  |  | 4.32 | 3.86 | 213 | 0.398 | 0.000 | 62, -44, 39 | Right Supramarginal Gyrus |
| Positive Interaction between C-Peptide AUC and Diagnosis | | |  |  |  |  |  |  |
|  |  | 4.62 | 4.13 | 281 | 0.179 | 0.000 | 57, -57, 36 | Right Angular Gyrus |
|  |  | 4.22 | 3.83 | 149 | 0.417 | 0.000 | 54, -46, 54 | Right Inferior Parietal Lobe |
| Negative Regression of GLP1 AUC and GMV (ND and AD) | | |  |  |  |  |  |  |
|  |  | 3.94 | 3.62 | 488 | 0.628 | 0.000 | 32, 18, -38 | Right Superior Temporal Gyrus |
| Positive Regression of CS Protein  and GMV (ND and AD) | | |  |  |  |  |  |  |
|  |  | 4.58 | 4.05 | 434 | 0.237 | 0.000 | -21, -8, 62 | Left Middle Frontal Gyrus |
|  |  | 3.98 | 3.61 | 179 | 0.673 | 0.000 | -44, 9, 57 | Right Middle Frontal Gyrus |
|  |  | 3.87 | 3.52 | 143 | 0.764 | 0.000 | -16, -69, 40 | Left Precuneus |
